# The role of wearable home blood pressure monitoring in detecting out-of-office control status

**DOI:** 10.1038/s41440-023-01539-w

**Published:** 2024-01-19

**Authors:** Heng-Yu Pan, Chih-Kuo Lee, Tzu-Yao Liu, Guan-Wei Lee, Chiao-Wei Chen, Tzung-Dau Wang

**Affiliations:** 1 Division of Cardiology, Department of Internal Medicine, National Taiwan University Hospital Hsinchu Branch, Hsinchu City, Taiwan; 2 Cardiovascular Center and Divisions of Cardiology and Hospital Medicine, Department of Internal Medicine, National Taiwan University Hospital and National Taiwan University College of Medicine, Taipei City, Taiwan

**Keywords:** Wearable device, Home blood pressure, Uncontrolled hypertension

## Abstract

Ambulatory blood pressure (ABP) and home blood pressure (HBP) monitoring is currently recommended for management of hypertension. Nonetheless, traditional HBP protocols could overlook diurnal fluctuations, which could also be linked with adverse cardiovascular outcomes. In this observational study, we studied among a group of treated hypertensive patients (*N* = 62, age: 52.4 ± 10.4 years) by using out-of-office ABP and wearable HBP. They received one session of 24-h ABP measurement with an oscillometric upper-arm monitor, and totally three sessions of 7-day/6-time-daily wearable HBP measurement separated in each month with HeartGuide. Controlled hypertension is defined as an average BP <130/80 mmHg for both daytime ABP and HBP. There was substantial reliability (intraclass correlation coefficient, ICC 0.883–0.911) and good reproducibility (Cohen’s kappa = 0.600) for wearable HBP measurement, especially before breakfast and after dinner. Among all patients, 27.4% had both uncontrolled HBP and ABP, 30.6% had uncontrolled HBP only, while 6.5% had uncontrolled ABP only. Female gender and increased numbers of anti-hypertensive agents are correlated with controlled hypertension. Patients with uncontrolled hypertension had a significantly higher maximal daytime blood pressure, which was previously signified as an imperial marker for cardiovascular risk. In conclusion, wearable HBP monitoring in accordance with a dedicated daily-living schedule results in good reliability and reproducibility. Patients with an uncontrolled wearable HBP should benefit from repeated HBP or ABP measurement for risk stratification.

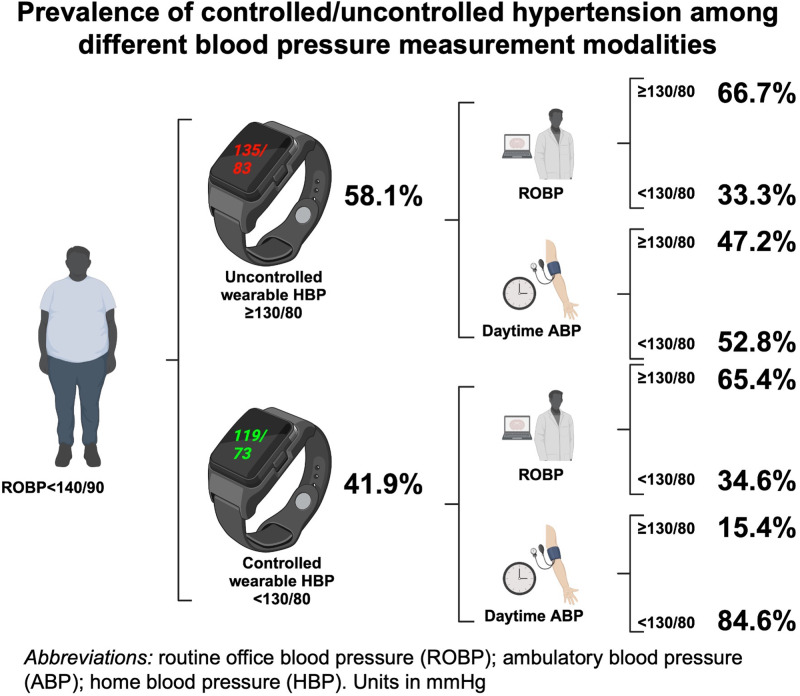

## Introduction

Out-of-office blood pressure (BP) monitoring is more emphasized by recently updated hypertension guidelines [1–4]. Uncontrolled hypertension detected by out-of-office modalities correlates with target organ damage more accurately than office readings [5, 6]. Both ambulatory blood pressure (ABP) and home blood pressure (HBP) modalities are complementary for hypertension management, considering their assessment in different BP profiles and inherent technical limitations. On one hand, ABP is measured frequently under various settings of activity including work, diet and sleeping, but it remains rather costly and bothersome to patients [7]. On the other, HBP is taken under standardized timing, which is only reliable under correct manual recordings and regular measurements [8, 9]. Diagnostic disagreement for uncontrolled hypertension between HBP and ABP varied from 8.2% to 18.3% [10, 11]. Meanwhile, the reproducibility of detecting uncontrolled hypertension by HBP is limited. The predictors of disagreement included anti-hypertensive treatment and office normotension [12].

In addition to average readings, daytime BP fluctuation is also an important issue. The consistent BP control is important, as more time spent in hypertensive range may be related to increased incidence of cardiovascular events [13]. Recently, more patients are tracking their health behaviors and vital readings on a regular basis with wearable devices [14]. Accumulating evidence revealed that smartwatches may assist to uncover subclinical cardiovascular disorders in ambulatory settings [15]. HeartGuide stands among the latest developed and validated wearable devices suitable for both out-of-office BP measurement and telemedicine applications [16]. In spite of the fact that wearable HBP readings measured by HeartGuide is in good agreement with ABP, reports of wearable HBP to detect control status of hypertension is scarce [17].

The following objectives are to be covered in this study. First, we studied the control status by wearable HBP and inspected predictors associated with controlled hypertension detected by wearable HBP. We also compared the status of BP fluctuation in between patients with or without controlled hypertension by HBP. Second, we investigated the reliability of wearable HBP measurement and the reproducibility to detect an elevated SBP at different daytime periods.

## Methods

### Study population

Patients actively receiving antihypertensive therapy with a routine office blood pressure (ROBP) <140/90 mmHg were recruited at the cardiovascular clinics at the National Taiwan University Hospital Hsin-chu Branch (Hsin-chu City, Taiwan). We excluded patients with terminal illness, end-stage renal disease, impaired performance status (ECOG ≥2), active pregnancy, resistant hypertension (actively taking ≥4 kinds of antihypertensive drugs), or known persistent arrhythmia. All participating patients provided written informed consent. This study was approved by the Institutional Review Board of the National Taiwan University Hospital Hsin-chu Branch (109-029-E). The study protocol was also registered at ClinicalTrials.gov (NCT04863508).

Demographic data, medications and laboratory data of all recruited participants were acquired from electronic health records. Echocardiography (EPIQ7 or IE33, GE Healthcare, Chicago, Illinois, United States) was performed by certified cardiologists. Left ventricular mass was calculated using the American Society of Echocardiography (ASE) formula [18, 19]. Left ventricular mass index (LVMI) was corrected by body surface area (BSA). Left ventricular hypertrophy (LVH) was defined as a female with an LVMI ≥95 g/m^2^ or a male with an LVMI ≥115 g/m^2^.

### Blood pressure measurement and variability index

The ROBP was obtained by using an automated oscillometric upper arm BP monitor while attended by registered nurses. BP measurement was taken with patients in a seated position after at least a 5-min rest before measurement. During a routine clinic visit, one ROBP reading each was required at 1^st^ and 3^rd^ month of the study respectively. Final report of the ROBP were obtained by the average of both readings.

ABP was measured for 24 h within the first month after recruitment with a validated oscillometric upper-arm BP monitor (BP3MZ1-1, Microlife Corporation, Taipei, Taiwan). Patients received measurements 30 min apart within the patient-defined awake/daytime period, and 60 min apart within the patient-defined asleep/nighttime period. Final report of 24-h, daytime and nighttime ABP were the average of all BP readings taken at respective timeframe.

**Fig. 1:**
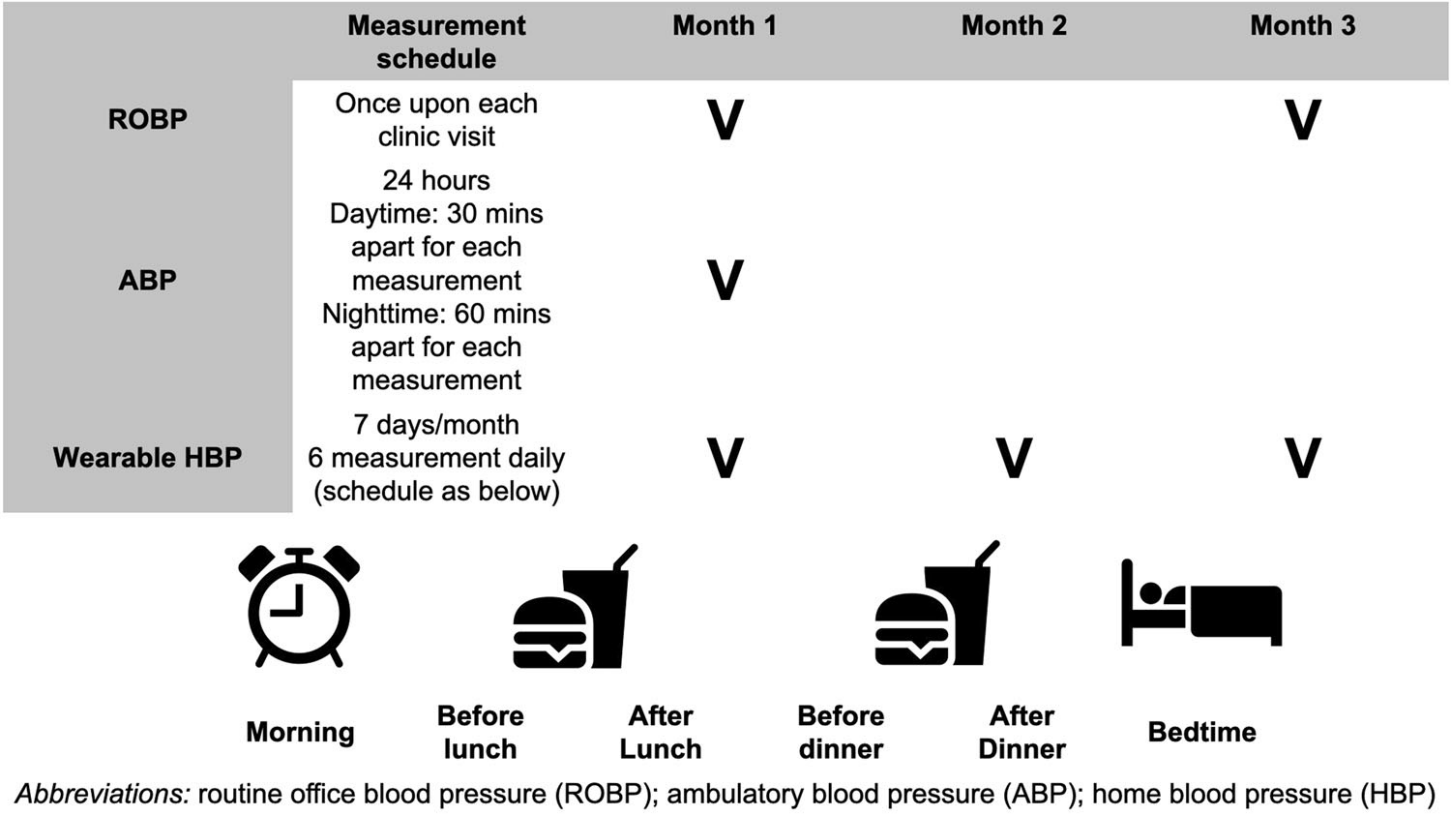
Measurement protocol for different modalities

Wearable HBP was measured by using HeartGuide (HEM-6411T-MAE; Omron Healthcare, Kyoto, Japan), a wrist-worn oscillometric BP monitor. Patients were instructed to wear the watch-type BP monitor at the non-dominant hand with positioning mark aligned to middle finger. They were required to sit quietly for 5 min beforehand, and to hold the device to heart level and two inches in front of the chest. Automatic BP measurement will then be taken at the appropriate position, which is confirmed by the built-in algorithm and notified with vibration of the device. One session is defined by HBP measurement by HeartGuide at six prespecified time periods daily for one week in each month. The session is repeated consecutively for 3 months. The prespecified periods include within 1 h after wakeup and before breakfast in the morning, 1 h before and after lunch, 1 h before and after dinner, and within 1-h before bedtime (Fig. 1). A total of 42 HBP readings is expected for each monthly session. All HBP readings were automatically accessed by the study team via BlueTooth transmission. The average wearable HBP was calculated for all monthly sessions (described as overall wearable HBP in the paragraph below), for each individual monthly session and for each individual measurement period.

The 2020 Taiwan Society of Cardiology/Taiwan Hypertension Society (TSOC/THS) Consensus on HBP recommended an ROBP <140/90 mmHg but HBP ≥135/85 mmHg be regarded as masked uncontrolled hypertension [1]. The 2022 TSOC/THS guideline had recently proposed a universal BP target <130/80 mmHg for ROBP, daytime ABP and wearable HBP, which is in agreement with the 2017 American College of Cardiology/American Heart Association (ACC/AHA) guideline [2, 4, 20]. Therefore, recruited patients with an ROBP <130/80 mmHg but an overall wearable HBP ≥130/80 mmHg is categorized as masked uncontrolled hypertension, and those with an ROBP ≥130/80 mmHg but an overall wearable HBP <130/80 mmHg is categorized as white-coat uncontrolled hypertension. Meanwhile, those with both ROBP and overall wearable HBP <130/80 mmHg are defined as sustained controlled hypertension, and those with both ROBP and overall wearable HBP ≥130/80 mmHg are defined as sustained uncontrolled hypertension.

The level of peak blood pressure was calculated as either the average of all wearable home SBP readings above 90^th^ percentile or the highest three readings. The BP variability were also calculated, as defined by coefficient of variation (CV) or average real variability (ARV).

### Statistical analysis

Between-group differences were verified using Student’s *t* test for continuous variables and Chi-square test for categorical variables. A stepwise multiple logistic regression analysis was performed for predictors associated with the presence of controlled hypertension by wearable HBP measurement. Additionally, the performance of wearable HBP measurement at different daytime periods was validated with inter-session reliability and reproducibility of detecting uncontrolled BP. Statistical significance was considered if a P value is <0.05.

The relatively reliability was shown by intra-class correlation coefficient (ICC) index, which is calculated by using one-way random-effects model of absolute agreement. An ICC index ≥0.75 indicates good reliability [21]. The absolute reliability was shown by Bradley-Blackwood test (F index) and repeatability coefficient (RC) [21, 22]. A nonsignificant F index means concordance of both means and variances among different monthly sessions. Meanwhile, RC demonstrates the precision of HBP measurement for all sessions, for which a lower RC value means a higher precision. Finally, the reproducibility of detecting an elevated SBP is shown by both percentage agreement and Cohen’s kappa value.

All analyses were carried out using Stata/MP (StataCorp, College Station, Texas, United States) and corresponding modules for calculating reliability (*icc*, *blandaltman*, *repeatability*) and reproducibility (*kappaetc*).

## Results

### Patient characteristics

**Fig. 2:**
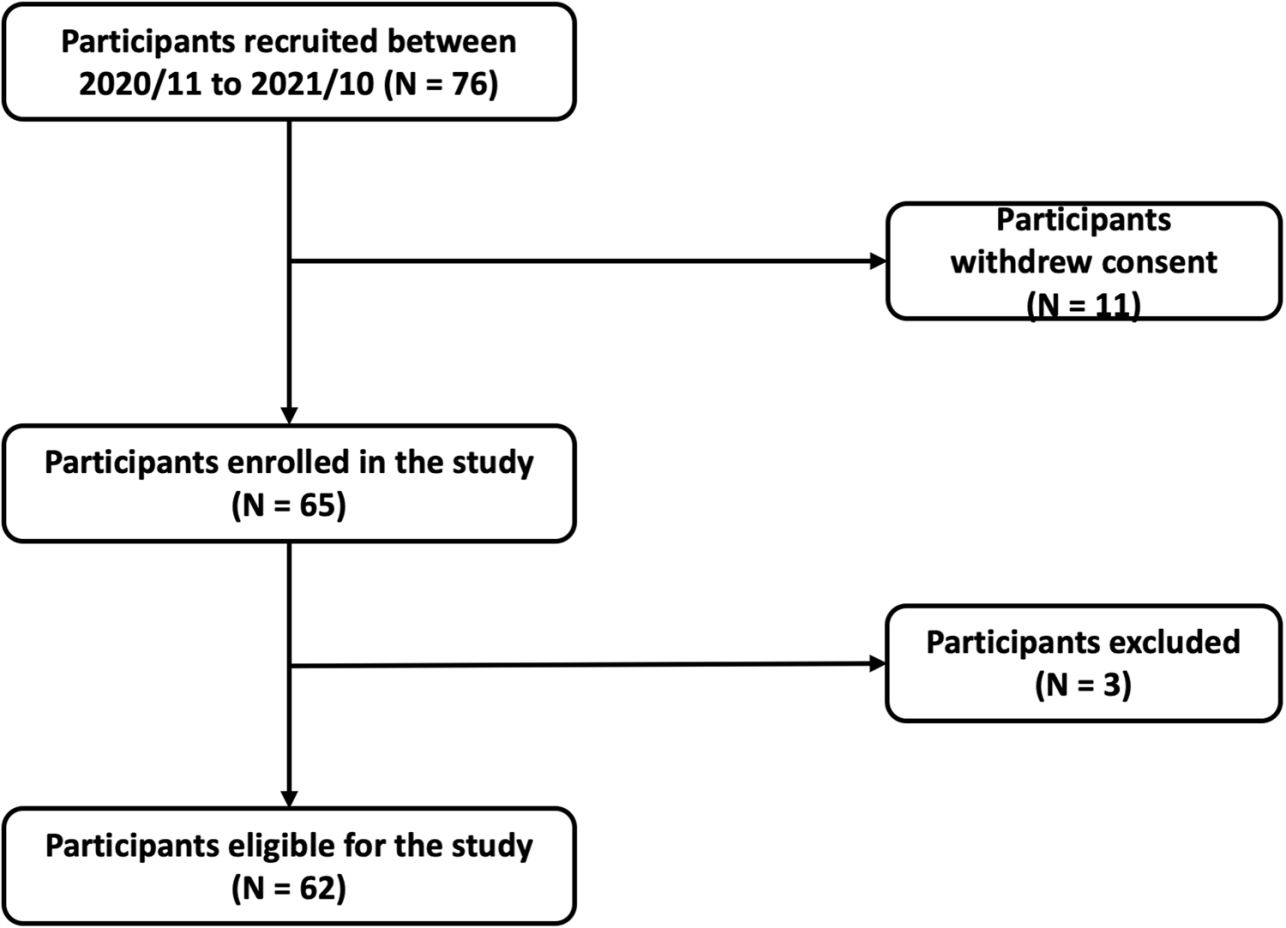
Flowchart for study participants

Patients treated for hypertension were recruited between November 2020 and October 2021. Overall, 76 patients initially provided informed consent, and 11 withdrew consent during the study period. An additional 3 patients were found to have uncontrolled ROBP or were unable to complete the study protocol, and therefore were excluded. Finally, 62 patients were included (44 men and 18 women) (Fig. 2). The average age was 52.4 ± 10.4 years, the average body mass index (BMI) was 26.5 ± 3.8 kg/m^2^, the average LVMI was 112.6 ± 24.7 g/m^2^. As for comorbidities, 12.9% of all patients had type 2 diabetes mellitus, 19.4% had coronary artery disease confirmed by angiography, and 3.2% had cerebrovascular disease. All patients used an average of 1.8 ± 0.7 anti-hypertensive drugs, with angiotensin receptor blockers (ARB) being most widely prescribed (79.0%), followed by calcium channel blockers (CCB) (45.2%) and beta blockers (37.1%).

### Blood pressure readings

All patients had an average count of 201.6 ± 95.3 wearable HBP readings within 3 months, and an average count of 30.7 ± 7.3 daytime ABP readings taken in first month. The average SBP was 130.9 ± 9.8 mmHg (ROBP), 118.6 ± 9.3 mmHg (24-h ABP), 121.6 ± 8.9 mmHg (daytime ABP), 112.6 ± 11.1 mmHg (nighttime ABP), and 128.6 ± 11.3 mmHg (overall wearable HBP) respectively. There is an average SBP difference of 10.0 ± 9.5 mmHg between 24-h ABP and overall wearable HBP, whereas the difference between daytime ABP and overall wearable HBP being 7.0 ± 9.7 mmHg (Supplementary Fig. 1a).

Meanwhile, the average DBP was 79.7 ± 7.7 mmHg (ROBP), 74.1 ± 6.9 mmHg (24-h ABP), by 76.0 ± 7.0 mmHg (daytime ABP), 70.3 ± 7.5 mmHg (nighttime ABP), and 78.9 ± 7.7 mmHg (overall wearable HBP). There is an average DBP difference of 4.8 ± 6.0 mmHg between 24-h ABP and overall wearable HBP, and the difference between daytime ABP and overall wearable HBP being 2.9 ± 6.4 mmHg (Supplementary Fig. 1b).

### Consistency of wearable HBP at different time periods

The reproducibility of detecting controlled hypertension by wearable HBP in each individual month was moderate (Cohen’s kappa = 0.600 between all 3 months of wearable HBP). The Bradley-Blackwood test (F index) showed concordance of HBP measurement at every pre-specified daytime period. The ICC was higher for HBP measurement taken in the morning, after dinner and before bedtime amongst all periods. The HBP measurement taken in morning, after dinner and before bedtime also had the lowest repeatability coefficient, which further suggested the relative consistency of readings at the above time periods. There is a higher percentage agreement and reproducibility regarding classification of elevated blood pressure based on readings taken in the morning and after dinner (Table 2).

### Overview and diagnostic agreement of control status

Thirty-six patients had uncontrolled hypertension by overall wearable HBP (58.1%). Patients with or without controlled hypertension by HBP had similar prevalence rates of major comorbidities and left ventricular hypertrophy, while those with controlled hypertension tended to be female and to be under more anti-hypertensive agents. Patients with controlled hypertension also had significantly lower 24-hour, daytime and nighttime ABP (Supplementary Table 1).

**Fig. 3:**
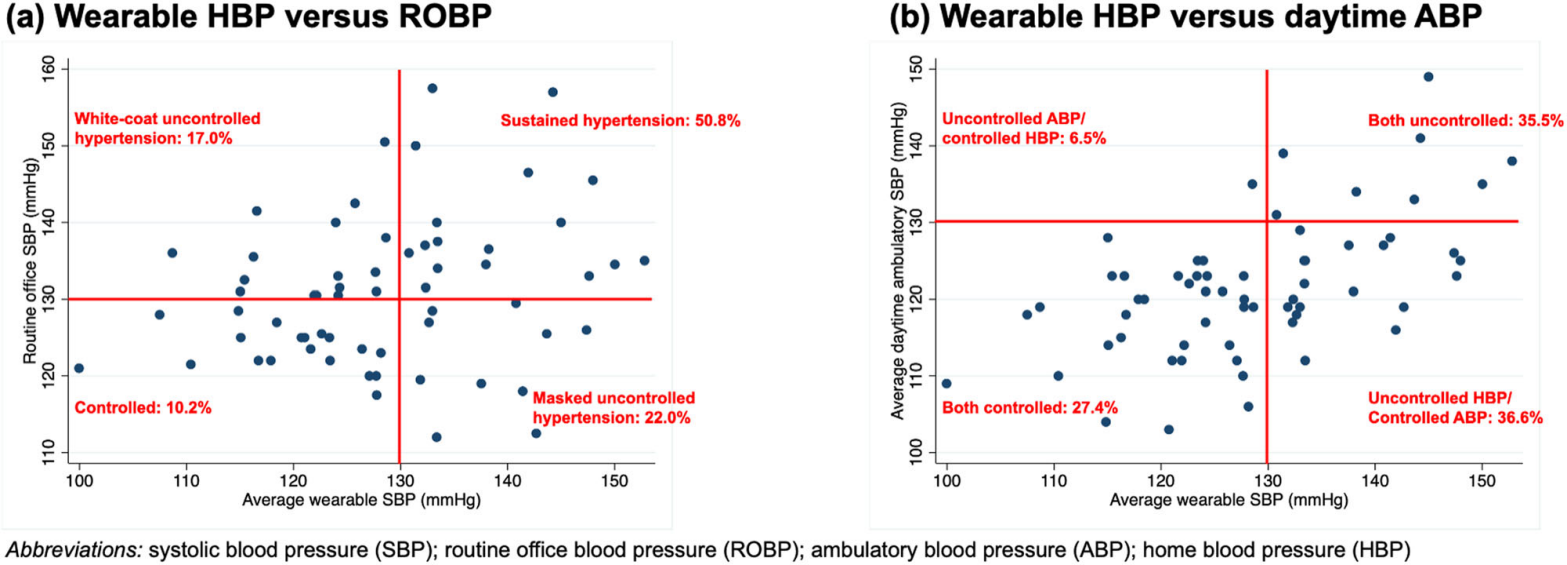
Comparison of control status between **a** wearable HBP versus ROBP; and **b** wearable HBP versus daytime ABP

When comparing the control status between both ROBP and wearable HBP measurement, 38.7% had sustained hypertension, 19.4% had masked uncontrolled hypertension, 27.4% had white-coat hypertension and finally 14.5% with sustained controlled hypertension (Fig. 3a). Meanwhile, 27.4% of all patients had uncontrolled hypertension for both daytime ABP and wearable HBP, 35.5% with controlled hypertension by both modalities, 30.6% with controlled daytime ABP but uncontrolled wearable HBP and 6.5% with controlled wearable HBP but uncontrolled daytime ABP (Fig. 3b).

**Fig. 4:**
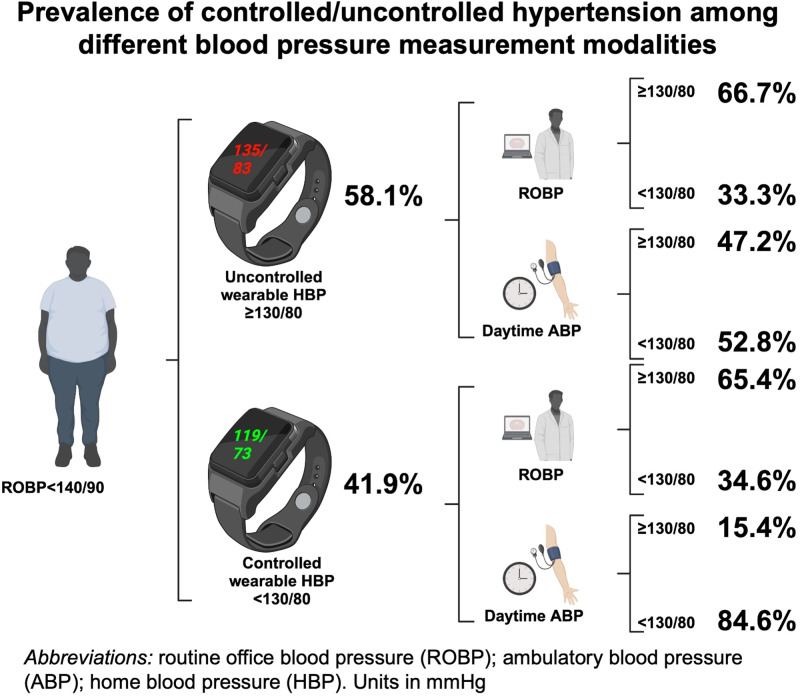
There is substantial agreement (84.6%) between wearable HBP and daytime ABP in terms of controlled hypertension. Female and those with more prescribed antihypertensive drugs are associated with controlled wearable HBP

In terms of control status, the overall agreement between daytime ABP and wearable HBP was good (the average of all HBP readings in overall sessions [percentage agreement: 62.9%] or the separate average of HBP readings in each individual monthly session [percentage agreement: 63.9–65.6%]). Most of patients (84.6%) with controlled wearable HBP also had controlled daytime ABP. Meanwhile, only half of all patients (47.2%) with uncontrolled wearable HBP also had uncontrolled daytime ABP (Fig. 4).

### Predictors of controlled hypertension with wearable HBP

Multiple logistic regression analysis was performed to identify predictors of uncontrolled hypertension by wearable HBP. The regression analysis was based on 2 models, first on all attributable clinical variables (model 1) and then focusing on variables with a *P* value <0.2 (model 2). All attributable clinical variables included age (<65 years old as reference), gender (male as reference), obesity (body mass index per 5 kg/m^2^ increase, with BMI <25 kg/m^2^ as reference), left ventricular hypertrophy, active smoking, history of coronary artery disease, history of diabetes, and number of prescribed anti-hypertensive drug. Amongst all, variables including gender, obesity, active smoking, history of coronary artery disease, history of diabetes, and number of prescribed anti-hypertensive drugs (monotherapy as reference) had P values below 0.2. These variables were analyzed in model 2. Female and more use of anti-hypertensive drugs are important predictors for controlled hypertension by wearable HBP. There was also a tendency towards controlled hypertension for patients without obesity, and those with diabetes (Table 3).

### Blood pressure variability by wearable HBP

The coefficient of variation for SBP was similar regardless of control status (controlled v. uncontrolled: 9.7 ± 2.3% v. 10.6 ± 2.2%, *P* = 0.131). There was also no difference in terms of average real variability for SBP (controlled v. uncontrolled: 11.9 ± 3.4 mmHg v. 11.6 ± 3.0 mmHg, *P* = 0.637). However, patients with uncontrolled hypertension had significantly higher peaked BP as measured by either the average SBP of all above 90^th^ percentile (controlled v. uncontrolled: 136.0 ± 9.4 mmHg v. 152.6 ± 9.8 mmHg, *P* < 0.001) or highest three readings (controlled v. uncontrolled: 150.8 ± 10.2 mmHg v. 166.6 ± 12.6 mmHg, *P* < 0.001).

## Discussion

To the author’s knowledge, this is the first study to investigate the prevalence of controlled hypertension by using a wearable BP device. Wearable HBP revealed good reliability for measurement and reproducibility in detecting the control status. Detecting the prevalence of an elevated SBP is more reproducible when HBP is measured in morning and after dinner than other time periods. Meanwhile, most of patients (84.6%) with well-controlled wearable HBP (<130/80 mmHg) also presented had well-controlled daytime ABP (<130/80 mmHg). Female gender and increased numbers of anti-hypertensive agents are associated with well-controlled status. Patients with uncontrolled hypertension had significantly higher peak daytime blood pressure.

Our study reported a relatively high reliability and good reproducibility of wearable HBP measurement over a relatively short time interval, particularly in the morning and after dinner. The preceding reports upon the performance of HBP monitoring are rather contradictory [23–25]. Some suggested that the reproducibility for HBP to unmask hypertension is suboptimal [26]. Specifically, post-prandial and bedtime BP readings are known to be influenced by meal content and bathing [27, 28]. Nonetheless, post-prandial BP is critical considering the magnitude of meal-related reduction is often associated with higher incidence of lacunar infarctions [29]. Repeated post-prandial HBP measurement may boost the reliability [30]. There are two unique features for this study. First, the study protocol requested to control the dosing regimen, dosing interval and measurement timing. Second, the dietary content for a typical dinner among most Taiwanese people could be generally consistent, while the content itself is highly influenced by socioeconomic background and dietary belief [31].

The percentage of agreement for controlled hypertension between wearable HBP and daytime ABP is higher than previous reports, though a nonnegligible difference of 7.0 ± 9.7/2.9 ± 6.4 mmHg exist between two modalities [11, 32–34]. The agreement in between ABP and HBP in each individual month remains similar. It is understandable as more daytime time-windows are considered by our protocol. Increased detection of uncontrolled hypertension by wearable HBP may be explainable due to sympathetic activation during daytime, and significantly fewer daily recordings compared with daytime ABP [35, 36]. Daytime BP, typically unveiled by ABP and often cloaked by conventional HBP, can predict the risk of cerebrovascular accidents [37]. Notably, around one-fifth of patients had changed controlled status in between monthly sessions, which basically reflects the inadequate reproducibility of out-of-office monitoring in a short time interval [38]. The importance of monthly measurement with wearable devices should therefore be emphasized. An increased number of anti-hypertensive medications is associated with better control, which is congruent with that most hypertensive patients require combination therapy to attain control [39]. A higher proportion of female patients showed adherence to medications, which could explain the superior control status among women patients compared with men [40].

Patients with an uncontrolled wearable HBP also had significantly higher peak daytime BP. In the presence of stiffened arteries, daytime peak BP may be more aggravated by stimuli such as work stress, smoking or temperature [41]. It was shown that a higher maximal daytime ABP is possibly associated with plaque rupture and ensuing stroke events [42, 43]. The findings from our study suggested the applicability of wearable HBP devices in guiding personalized anti-hypertensive treatment, specifically with the target of ameliorating BP fluctuation [44].

Our study has some limitations. First of all, the number of recruited participants by our initial plan was 90 to prove the reproducibility of detecting uncontrolled hypertension. The course of this study was hampered by the strike of COVID pandemic. Nonetheless, substantial agreement between HBP sessions was still observed. Secondly, ABP and wearable HBP were not performed on the concurrent timing. The comparison between both modalities may be flawed by unadjusted lifestyle factors. However, most enrolled patients were office workers, and their daily-living schedule may remain unaltered during the study period. Third, drinking habit could affect the evening HBP, but was not included in our collected demographic information. Nonetheless, patients with habitual drinking are actually expected to be minority. Only 5.1% of all Taiwanese adults reported habitual drinking to the inquiry questionnaire during May Measurement Month campaign in 2017 [9]. Finally, this was a cross-sectional study in which unidentified confounders could likely occur. Still, the prevalence rates of all identified demographic factors and comorbidities were similar between those with and without controlled HBP.

In conclusion, wearable HBP yielded good reliability and reproducibility for out-of-office monitoring, especially with measurements taken in morning and after dinner. Female gender and higher number of anti-hypertensive agents are associated with uncontrolled hypertension. There is significant agreement between wearable HBP and daytime ABP with a BP level <130/80 mmHg. Patients with an elevated wearable HBP should receive repeated out-of-office monitoring for diagnostic confirmation.

### Supplementary information

Supplementary information
