# Supplementary material for: The role of wearable home blood pressure monitoring in detecting out-of-office control status
Source: Hypertens Res. 2024 Jan 19;47(4):1033–41. doi: 10.1038/s41440-023-01539-w (PMC10994837; doi:10.1038/s41440-023-01539-w)
Supplement: Supplementary file 1 — Supplementary information [file 41440_2023_1539_MOESM1_ESM.docx]

**Supplementary table 1. Comparison of patient characteristics with or without controlled hypertension**

| **Clinical variables** | **Uncontrolled hypertension by wearable HBP**  **(N=36)** | **Controlled hypertension**  **by wearable HBP**  **(N=26)** | **P value** |
| --- | --- | --- | --- |
| Age (years) | 52.6±10.3 | 52.2±10.6 | 0.896 |
| Female, No (%) | 7(19.4) | 11(42.3) | **0.050** |
| Active smoker, No (%) | 7(19.4) | 3(11.5) | 0.377 |
| BMI (kg/m^2^)* | 26.7±3.9 | 26.2±3.8 | 0.658 |
| Diabetes mellitus, No (%) | 3(8.3) | 5(19.2) | 0.207 |
| CAD, No (%) | 8(22.2) | 4(15.4) | 0.501 |
| Stroke, No (%) | 2(5.6) | 0 |  |
| Serum creatinine (mg/dL)* | 1.1±0.4 | 0.9±0.2 | 0.082 |
| Total cholesterol (mg/dL) | 177.1±53.7 | 184.6±43.6 | 0.623 |
| LDL-C (mg/dL) | 103.3±35.3 | 106.6±34.2 | 0.735 |
| Triglyceride (mg/dL)* | 161.6±109.2 | 162.5±68.1 | 0.969 |
| Glycated hemoglobin (%)* | 5.9±1.4 | 5.8±0.5 | 0.841 |
| LVMI (gm/m^2^) | 110.8±26.9 | 115.2±21.4 | 0.498 |
| LVH, No (%) | 17(47.2) | 17(65.4) | 0.134 |
| Numbers of anti-hypertensive agents | 1.6±0.8 | 2.0±0.6 | **0.015** |
| ARB, No (%) | 26(72.2) | 23(88.5) | 0.121 |
| Beta-blockers, No (%) | 11(30.6) | 12(46.2) | 0.210 |
| CCB, No (%) | 13(36.1) | 15(57.7) | 0.092 |
| Diuretics, No (%) | 2(5.6) | 1(3.9) | 0.757 |
| Alpha blockers, No (%) | 4(11.1) | 1(3.9) | 0.300 |
| Others, No (%) | 3(8.3) | 1(3.9) | 0.478 |
| Office SBP (mmHg) | 131.7±11.3 | 129.9±7.2 | 0.486 |
| Office DBP (mmHg) | 79.8±8.1 | 79.5±7.1 | 0.899 |
| 24-hour ambulatory SBP (mmHg) | 121.6±9.1 | 114.4±7.9 | **0.002** |
| 24-hour ambulatory DBP (mmHg) | 76.6±6.0 | 70.6±6.5 | **<0.001** |
| Daytime ambulatory SBP (mmHg) | 124.6±8.7 | 117.5±7.5 | **0.001** |
| Daytime ambulatory DBP (mmHg) | 78.4±6.4 | 72.8±6.6 | **0.001** |
| Nighttime ambulatory SBP (mmHg) | 115.3±10.9 | 108.7±10.6 | **0.020** |
| Nighttime ambulatory DBP (mmHg) | 72.9±6.6 | 66.8±7.3 | **0.001** |
| Wearable home SBP (mmHg) | 135.3±8.7 | 119.3±7.3 | **<0.001** |
| Wearable home DBP (mmHg) | 83.2±6.3 | 73.0±5.1 | **<0.001** |
| BMI: body mass index; LVMI: left ventricular mass index; LVH: left ventricular hypertrophy; CAD: coronary artery disease; ARB: angiotensin receptor blocker; CCB: calcium channel blocker; HBP: home blood pressure; SBP: systolic blood pressure; DBP: diastolic blood pressure  *not normally distributed | | | |

**Supplementary Figure 1. Bland-Altman plots for both wearable HBP and daytime ABP**

**
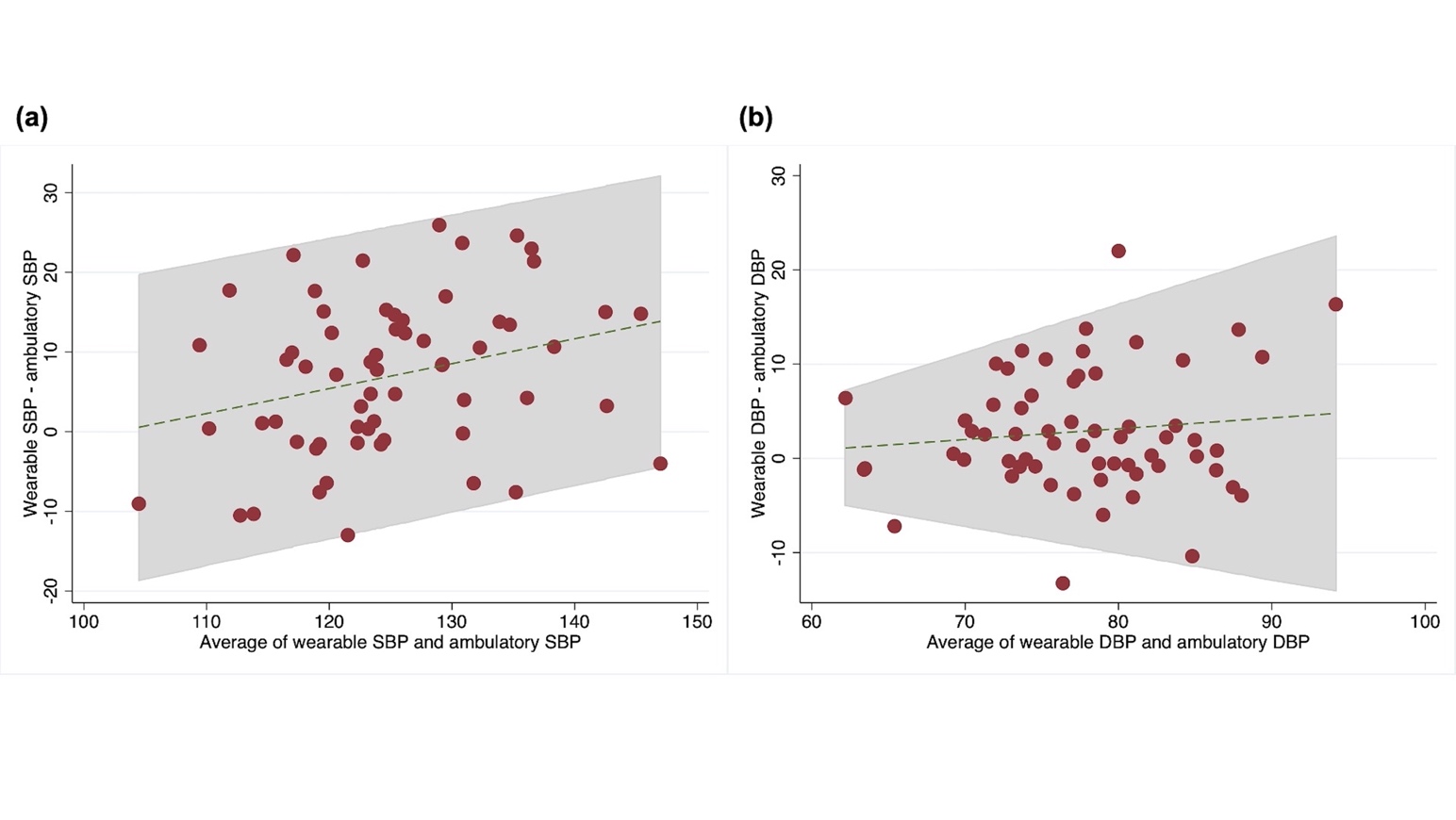
**
